# Catalepsy

**Published:** 1889-08

**Authors:** 


### CATALEPSY.

There is no marked change in one during an attack of catalepsy. The heart still beats, and the lungs are doing their work ; in fact, all the general vital functions are going on. In some cases, however, all the vital organs are depressed and do their work feebly. Respiration is then slower, and may even, in very severe attacks, become imperceptibe. The heart also shares in the loss of power, and its movements may be so weak, that pulsation in the arteries cannot be detected. As for the temperature of the body, that generally remains the same as in health : but in cases where the lungs and heart are enfeebled, as we have described, it sinks two or three degrees below the normal, and the skin is icy cold and clammy. In these cases, of course, the victim of the fit has really the appearance of death. Eulenburg says very severe cases, in which, with icy cold skin, the pulse and respiration became so weak as to be imperceptible, may have led the laity to consider the patients apparently or truly dead. Such cases form the foundation for the many exaggerated, and the wonderfully distorted stories in regard to cataleptic persons who have seemed to be dead during several days, or who have been buried alive. It is perfectly natural that the above phenomena, as also the wonderful peculiarities of the caleptic condition, should always make a specially wierd impression upon the laity, and thus give material for exaggeration and invention. But all such stories, concerning apparent death and burying alive, are certainly so distorted and misrepresented that even in the most favorable case it is difficult to discover the grain of truth upon which they are founded. But more recently there are cases on record which show the possibility of such occurrences.

Now to the matter of diagnosis. Ought it to be difficult to distinguish between the cataleptic state and death ? Except in very rare instances it ought not to be difficult. Is it impossible to distinguish between that state and death in the most severe cases ? To this question the answer no, can be unhesitatingly given. It is true that the only positive sign of death is decomposition ; but there are a number of tests held to be quite reliable, and if several of these are applied in a case always with negative results, but little doubt can exist. But no conscientious physician will ever take chances in a case of this sort where there exists the slightest doubt. He would insist that burial be delayed until all trace of it was removed. Some old and reliable authorities in medicine have told of cases where cataleptics have been buried and returned to life again ; we can scarcely doubt that such were authentic. But in this connection there is one important point to be considered. Medical science has made rapid advances in the many years that have elapsed since the last of these cases was reported. It cannot be so very long ago when the one test of death almost wholly relied upon was the looking glass held to the mouth. But at the present time there are twenty, perhaps thirty, tests, every one of which is even more reliable than that old one. Physicians of to-day are infinitely better equipped and made far more discerning by a higher education than those of even a half a century ago. This danger of burial before death has ever been fully appreciated by them, and how to prevent such a terrible accident has been a constant study with them. Always conscious of the danger, it is scarcely possible that a physician —in all that the name implies—would ever allow it to happen.

